# Cigarette Smoking Blocks the Protective Expression of Nrf2/ARE Pathway in Peripheral Mononuclear Cells of Young Heavy Smokers Favouring Inflammation

**DOI:** 10.1371/journal.pone.0008225

**Published:** 2009-12-09

**Authors:** Ulisse Garbin, Anna Fratta Pasini, Chiara Stranieri, Mattia Cominacini, Andrea Pasini, Stefania Manfro, Fabio Lugoboni, Chiara Mozzini, GianCesare Guidi, Giovanni Faccini, Luciano Cominacini

**Affiliations:** 1 Department of Biomedical and Surgical Sciences, Section of Internal Medicine, University of Verona, Verona, Italy; 2 Medical Service for Addictive Disorders, University Hospital G. B. Rossi, Verona, Italy; 3 Department of Morphological and Biomedical Sciences, Section of Chemistry and Clinical Microscopy, University of Verona, Verona, Italy; Fundação Oswaldo Cruz, Brazil

## Abstract

Cigarette smoking is an important risk factor for atherosclerosis, a chronic inflammatory disease. However the underlying factors of this effect are unclear. It has been hypothesized that water-soluble components of cigarette smoke can directly promote oxidative stress in vasculature and blood cells. Aim of this study was to study the relationship between oxidative stress and inflammation in a group of young smokers. To do this we evaluated: 1) the oxidation products of phospholipids (oxPAPC) in peripheral blood mononuclear cells (PBMC); 2) their role in causing PBMC reactive oxygen species (ROS) generation and changes in GSH; 3) the expression of the transcription factor NF-E2-related factor 2 (Nrf2) and of related antioxidant genes (ARE); 4) the activation of NF-kB and C-reactive protein (CRP) values. We studied 90 healthy volunteers: 32 non-smokers, 32 moderate smokers (5–10 cigarettes/day) and 26 heavy smokers (25–40 cigarettes/day). OxPAPC and p47phox expression, that reasonably reflects NADPH oxidase activity, were higher in moderate smokers and heavy smokers than in non-smokers (p<0.01), the highest values being in heavy smokers (p<0.01). In *in vitro* studies oxPAPC increased ROS generation via NADPH oxidase activation. GSH in PBMC and plasma was lower in moderate smokers and heavy smokers than in non-smokers (p<0.01), the lowest values being in heavy smokers (p<0.01). Nrf2 expression in PBMC was higher in moderate smokers than in non-smokers (p<0.01), but not in heavy smokers, who had the highest levels of NF-kB and CRP (p<0.01). In *in vitro* studie*s* oxPAPC dose-dependently increased NF-kB activation, whereas at the highest concentrations Nrf2 expression was repressed. The small interference (si) RNA-mediated knockdown of NF-κB/p65 increased about three times the expression of Nrf2 stimulated with oxPAPC. Cigarette smoke promotes oxPAPC formation and oxidative stress in PBMC. This may cause the activation of NF-kB that in turn may participate in the negative regulation of Nrf2/ARE pathway favouring inflammation.

## Introduction

Atherosclerosis is a chronic inflammatory disease of the arterial wall [1]–[3] with enormous epidemiological relevance [4], [5]. Sound evidence has been generated that oxidative stress is one of the most potent inductor of vascular inflammation in atherogenesis [6]. Reactive oxygen species (ROS) are known to change the oxidation-reduction (redox) state of the exposed cells and it is known that several inflammatory genes and the related transcription factors are regulated through redox-sensitive mechanisms [1]. Nuclear factor (NF)-kB was the first eukaryotic transcription factor shown to respond directly to oxidative stress. A huge amount of experimental data supports the activation of the transcription factor NF-kB as a key redox-sensitive event associated with vascular dysfunction (reviewed in 7). NF-kB intervenes in the transcription of a large number of inflammatory genes coding for cytokines, chemokines, and adhesion molecules [8].

Cigarette smoking is the worldwide leading cause of preventable morbidity and mortality and constitutes a major risk factor for atherosclerotic vascular disease, including stroke and coronary artery disease [9]. Cigarette smoke can be divided into two phases: tar and gas-phase smoke. Both phases contain high concentrations of ROS, nitric oxide, peroxynitrite, and free radicals of organic compounds [10], [11]. In addition to these short-lived, highly reactive substances, previous studies have shown that aqueous cigarette tar extracts also contain pro-oxidant substances that have the potential to increase cellular production of ROS [12]. Thus it has been hypothesized that water-soluble components of cigarette smoke that are likely to reach the systemic circulation can directly promote oxidative stress in vasculature and blood cells [10], [12].

The phospholipid 1-palmitoyl-2-arachidonyl-sn-glycero-3-phosphorylcholine (PAPC) is a major component of cell membranes and lipoproteins. Oxidation products of PAPC (oxPAPC) are found in cells during inflammation, in membranes of apoptotic cells, as well as in oxidized low density lipoprotein and are considered sensitive markers of oxidative stress (reviewed in 13). Furthermore ox-PAPC have been shown to induce ROS production in vascular cells that appears to be mediated largely by NADPH-oxidase activity [14].

The antioxidant response element (ARE), also referred to as the electrophile response element, is a cis-acting transcriptional regulatory element involved in the activation of genes coding for a number of antioxidant proteins and phase II detoxifying enzymes including NADPH quinone oxidoreductase, glutathione peroxidase, ferritin, gamma-glutamylcysteine synthetase (gGCS ) and heme oxygenase-1 (HO-1) [15]. NF-E2-related factor 2 (Nrf2) is the transcription factor that is responsible for both constitutive and inducible expression of the ARE-regulated genes [15]. Nrf2-null mice have decreased basal and inducible expression of antioxidant genes, increased oxidative stress, and decreased reducing activity and antioxidant capacity [16], suggesting that the Nrf2/ARE pathway is critical for the regulation of intracellular redox status. Recently it has been shown that NF-kB p65 may participate in the negative regulation of Nrf2/ARE signalling [17].

Since cigarette smoking is a risk factor for atherosclerosis that promotes oxidative stress and oxidative stress has been shown to induce the redox sensitive transcription factor NF-kB, as well as the counteracting Nrf2/ARE pathway, we aimed this study to evaluate the balance between these two inducible processes in blood cells derived from young healthy smokers and the effects on systemic indices of inflammation.

## Methods

### Participants

The study was approved by the Ethical Committee of University of Verona and all participants provided written consent prior to commencing the study.

Healthy volunteers were recruited among the students of Verona University and among nurses of local Universitary Hospital. A group of heavy smokers was also recruited from the regional center for smoking. The healthy volunteers were recruited according to the following criteria: male and female; aged 18–35 years; BMI 19–28 kg/m^2^; drink less than 5 units of alcohol per week, with 1 unit equivalent to 7.9 g alcohol; should not be following a weight reducing diet, exercise no more than 3×30 min aerobic exercise per week; no use of antioxidant supplementation or anti-inflammatory medication during or for six months prior to commencing the study; no diagnosed heart disease, diabetes or endocrine disorder. Smokers were described as individuals who smoked 5–10 (moderate smokers) or 25–40 (heavy smokers) cigarettes per day for at least 3 years, whilst non-smokers included those who had never smoked or those who had not smoked for at least 3 years.

### Blood Samples and Peripheral Blood Mononuclear Cells (PBMC) Isolation

Venous blood samples were obtained from volunteers after 12 h fasting.

Blood was collected from each subject and drawn into pyrogen-free blood collection tubes. Multiple aliquots of serum or plasma were placed into sterile 1-ml screw-capped polypropylene vials, containing phenolic antioxidant 2,6-Di-tert-butyl-4-methylphenol (BHT) (10 mM) (SIGMA) to inhibit lipid peroxidation, and stored at −80°C. Samples were kept frozen for no longer than 9 months with an average of 5 months. The samples were frozen and thawed only once. PBMC were isolated as previously described [18]. Briefly whole blood was layered onto a sterile aqueous medium containing ficoll and sodium diatrizoate at a predetermined density of 1.007 g/ml at 25°C. Gentle centrifugation at room temperature resulted in the separation of PBMC at the blood/ficoll interface, with the other white and red blood cells passing through the interface. Total cholesterol, high density lipoprotein (HDL) cholesterol, LDL cholesterol, triglycerides, glucose, white blood cell (WBC) count, were measured with standard methods. C-reactive protein (CRP) was measured by a commercially available high sensitivity turbidimetric method (Syncron-PCR, Beckman). Interleukin- (IL) 6 in plasma was measured with a commercial assay kit (Quantikine, R&D System).

### Evaluation of oxPAPC in PBMC and Plasma from Non-Smokers, Moderate Smokers and Heavy Smokers

OxPAPC in PBMC and plasma of non-smokers and smokers were measured on an Agilent mass spectrometer equipped with an electrospray source as previously described [19]. The following different oxPAPC were taken into consideration: 1-palmitoyl-2-(5,6-epoxyisoprostane E2)-sn-glycero-3-phosphocholine (PEIPC); 1-palmitoyl-2-(5-oxovaleroyl)-sn-glycero-3-phosphorylcholine (POVPC); 1-palmitoyl-2-glutaroyl-sn-glycero-3-phosphorylcholine (PGPC). Flow injection experiments were performed by an HPLC system (HP1100; Agilent Technologies). Quantification of the peak areas was performed by single ion monitoring in the elution time range of 10–20 min using appropriate software.

Authentic1-palmitoyl-2-arachidonoyl-sn-glycero-3-phosphocholine (PAPC), POVPC and PGPC were obtained from Avanti Polar Lipids, Inc. (Alabaster, AL). PEIPC was prepared and analyzed in our laboratory as previously described [20].

### Quantitative Real-Time PCR

Quantitative Real-Time PCR analysis was performed as previously described [18]. Total RNA was extracted from PBMC of moderate smokers, heavy smokers and non-smokers with a RNeasy Mini Kit (Qiagen) and was reverse transcribed using IScript cDNA Synthesis Kit (Bio-Rad, Hercules, CA). The relative expression levels of mRNA encoding Nrf2, HO-1, IkB-alpha, gGCS, p47phox, p65, IL-6 and beta-actin were measured by iCycler (Bio-Rad, Hercules, CA), using IQSYBR Green PCR SuperMix (Bio-Rad, Hercules, CA) and 300 nM each primer pair. Primers were designed by Beacon Design 4.0 software (PREMIER Biosoft International, Palo Alto, CA, USA) and synthesized by MWG Biotech AG (Ebersberg, Germany) (Nrf2, sense 5′-TTCAGCCAGCCCAGCACATC-3′ and antisense 5′-CGTAGCCGAAGAAACCTCATTGTC-3′; HO-1, sense 5′-GGTGACCCGAGACGGCTTC-3′ and antisense 5′-AGACTGGGC TCTCCTTGTTGC-3′; IkB-alpha, sense 5′-CGCACCTCCACTCCATCCTG-3′, antisense 5′-GCATTGACATCAGCACCCAAGG-3′; gamma-glutamylcysteine synthetase (gGCS) (heavy chain), sense 5′ CGAATTCGCCAAGAATGAGGAGATCC -3′, antisense 5′- CGAATTCGAAAGCGACGGCTGTACC-3′; p47phox, sense 5′-CCCACAGACAACCAG ACAAA-3′, antisense 5′-TTTGTCTGGTTGTCTGTGGG-3; p65, sense 5′-GGCCATGGACGAACT GTTCCC-3′, antisense GGAGGGTCCTTGGTGACCAG-3′; IL-6, sense 5′-GCGCCTTCGGTCCAGTTG-3′, antisense 5′-CTGCTTTCTCAGGGCTGAG 3′; β-actin, sense 5- ATCAAGATCATTGCTCCTCCTG-3 and antisense 5′- GCAACTAAGTCATAGTCCGCC-3′). Normalized gene expression levels were given as the ratio between the mean value for the target gene and that for the beta-actin in each sample.

### Western Blot Analysis

Western blot analysis was performed as previously described [18]. Nrf2, HO-1, gGCS, IkB-alpha, p47phox and p65 were immunoprecipitated from 1 mg of each PBMC protein lysate or nuclear lysate with mouse monoclonal anti-Nrf2 (437C2a), anti HO-1 (HO-1-1), anti-gamma-GCS (LL-8), anti-IkB-alpha (H-4), anti-p47phox (A-7), anti-p65 (340C1a), anti IL-6 (A00042.01) antibodies, and anti-bet-actin (Santa Cruz Biotechnology). Immune complexes were captured with protein A/G-Sepharose beads (Pierce) for 2 h at 4°C, and the beads were washed four times with 100 mM NaCl. To ensure the specificity of protein-antibody interaction, lysates were incubated with beads in the absence of antibody as well as with an irrelevant immunoglobulin isotype control (Caltag). Nrf2, HO-1, gGCS, IkB-alpha, p47phox and p65 were detected by probing immunoprecipitates with rabbit polyclonal anti Nrf2 (C-20) (sc-722), anti HO-1 (H105) (sc-10789), anti gGCS (H-300) (sc-22755), anti IkB-alpha (C-15) (sc-203), anti p47phox (H-195) (sc-14015), anti p-65 (A) (sc-109), anti IL-6 (H-183) (sc-7920) and beta-actin (N-21) (sc-130656) antibodies (Santa Cruz Biotechnology), followed by goat anti-rabbit horseradish peroxidase-conjugated secondary antibody (Bio-Rad). Reactive antigens were visualized with Supersignal chemiluminescence substrate (Pierce) at about 110 (Nrf2), 32 (HO-1), 73 (gGCS), 37 (IkB-alpha), 47 (p47phox), 65 (p65), 26 (IL-6) and 43 (beta-actin) kDa and quantified by densitometric analysis with ChemiDoc XRS (Bio-Rad). Protein expression data was quantified with Quantity One Software (Bio-Rad). As positive control tert-butylhydroquinone (t-BHQ) (100 µM) (Sigma-Aldrich) and phorbol myristate acetate (PMA) (100 nM) (Sigma-Aldrich) were used respectively for Nrf2, HO-1, gamma-GCS and for p47phox, IkB-alpha, p65, NF-kB and IL-6.

### Measurement of NF-kB Activation in Isolated PBMC

Nuclear factor-kB activation was measured by a sensitive multi-well colorimetric assay for nuclear NF-kB (TRANS-AM; Active Motif, Rixensart, Belgium), as previously described [21]. Nuclear extract was obtained by utilizing the nuclear extract kit supplied by Active Motif. As a reference, recombinant p65 (Active Motif) was used.

### GSH Measurement in PBMC and Plasma

The detailed procedures for the measurements of cellular and plasma GSH have been previously described [22]. Samples were collected directly into specially prepared tubes containing preservative BHT (10 mM) to reduce auto-oxidation, and frozen at −80°C. Samples were analyzed using high-performance liquid chromatography with fluorescence detection of 7-fluorobenzo-2-oxa-1,3-diazol-4-sulfonic acid (SBD-F) at excitation 385 nm and emission 515 nm.

### Effect of Purified oxPAPC on ROS Generation, on Expression of Nrf2/ARE Pathway and on NF-kB Activation in PBMC of Healthy Donors

Purified monocytes (3×10^5^/ml, 200 µl/well) of healthy donors were cultured in RPMI 1640 containing L-glutamine (GIBCO) for 20 hours at 37°C with increasing amounts (from 8 to 32 µM) of POVPC, PGPC and PEIPC.

Some experiments were performed in the presence of the inhibitors of NADPH oxidase apocynin (50 µM) and diphenyliodonium (DPI) (50 µM), vitamin C (0–400 µM) and after small interference (si) RNA-mediated knockdown of p47phox, Nrf2 and p65. ROS were measured as described [23]. Cell viability was monitired by using 7-amino-actinomycin D (7-AAD)-(BD Biosciences) [24]. Endotoxin contamination of cell cultures was routinely excluded with the chromogenic Limulus amebocyte lysate assay (Sigma).

### siRNA-Mediated Knockdown of p47phox, Nrf2 and p65

Cells were transfected with siRNA against p47phox (5′-TTTGTCTGGTTGT TGTGGG-3′), or scrambled (sc) siRNA (Ambion, Austin, TX), against Nrf2 siRNA (5′-GUUUUUCCAGCUCAUACUCUUTT-3′), or negative control (Invitrogen) and against p65 (5′-GAUCAAUGGCUACACAGGA-3′), or negative control (GenPharma) as previously described [25].

### Statistical Analysis

Data are expressed as means ± SD values if normally distributed. Normal distribution of the data was determined using Shapiro-Wilk test. Differences between continuous data were analysed by two-tailed unpaired Student's t-test. Statistical comparison between 3 groups was performed by one-way ANOVA and post-hoc multiple comparison using Student-Newmann-Keuls' test. Relationship between variables was assessed by linear regression. A probability value (p)<0.05 was considered to be statistically significant. All data were analyzed with SPSS 11.04 for Macintosh (SPSS, Chicago, III.).

## Results

### Clinical Characteristics of the Subjects

Along a period of 6 months, 124 healthy volunteers were invited to participate to the study; of these only 90 (32 non-smokers, 32 moderate smokers and 26 heavy smokers) fully satisfied the enrolling criteria. Clinical and metabolic characteristics of the subjects are described in Table 1. There were no statistical differences among the clinical and metabolic data in non-smokers, moderate smokers and heavy smokers.

**Table 1:** Clinical and metabolic characteristics of non-smokers, moderate smokers and heavy smokers.

	NON-SMOKERS (n = 32)	MODERATE SMOKERS (n = 32)	HEAVY SMOKERS (n = 26)	P
Age	25.3±3.4	26.7±3.9	30.3±3.6	ns
Male/female	16/16	18/14	12/14	ns
BMI (Kg/m2)	22.6±2.6	23.7±3.7	22.8±3.1	ns
SBP (mmHg)	111.7±12.6	113.2±9.1	114.3±8.5	ns
DBP (mmHg)	73.9±12.2	74.6±8.6	75.3±8.2	ns
Heart rate (bpm)	66.6±9.6	69.7±8.0	71.3±7.8	ns
Waist circumference (cm)	78.0±10.8	76.3±11.4	76.8±10.4	ns
Total cholesterol (mg/dl)	190.7±29.6	184.8±34.7	188.4±22.3	ns
LDL cholesterol (mg/dl)	109.5±26.1	106.8±26.9	104.3±19.7	ns
HDL cholesterol (mg/dl)	53.5±10.2	57.4,±11.2	54.4±10.9	ns
Triglycerides (mg/dl)	84.5±38,5	87.2±47,6	94.3±34.3	ns
Plasma glucose (mg/dl)	72.7±10.4	73.1±7.5	75.3±8.2	ns

BMI = body mass index; SBP = systolic blood pressure; DBP = diastolic blood pressure.

### Inflammatory Markers and GSH Concentrations

Moderate smokers and heavy smokers had significantly higher number of WBC, neutrophils, levels of circulating IL-6 and CRP (p<0.01) and lower GSH concentrations (both in PBMC and plasma) than non-smokers (p<0.01) (Table 2). These inflammatory markers resulted significantly higher and cellular and plasma GSH levels significantly lower in heavy smokers than in moderate smokers (p<0.01).

**Table 2:** Inflammatory markers and GSH concentrations in non-smokers, moderate smokers and heavy smokers.

	NON-SMOKERS (n = 32)	MODERATE SMOKERS (n = 32)	HEAVY SMOKERS (n = 26)	P
WBC (mmc)	5279.6±965.3	6576.8±1670.4*	7800.8±881*†	<0.01
Neutrophils (mmc)	2825.3±751.7	3713.7±1441.6*	4821.8±754*†	<0.01
Lymphocytes (mmc)	1856.6±365.3	2186.4±656.5	2024.5±411	ns
Monocytes (mmc)	371.7±171.0	436.9±168.5	411.7±181	ns
IL-6 (pg/ml)	0.32±0.041	0.68±0.066	1.54±0,21	<0.01
CRP (mg/dl)	0.06±0.007	0.13+0.015*	0.35+0.033*†	<0.01
PBMC GSH (ng/mg cell protein)	2.4±0.22	1.9±0.21*	1.1±0.13*†	<0.01
Plasma GSH (µmol/L)	6.8±0.71	5.1±0.47*	3.2±0.38*†	<0.01

WBC = white blood count; IL-6 = interleukin-6; CRP = high-sensitivity C reactive proten; PBMC = peripheral blood mononuclear cells. Data are expressed as means±SD; *P<0.01 vs non-smokers; †P<0.01 vs moderate smokers.

### OxPAPC Concentrations in PBMC and Plasma of Non-Smokers, Moderate Smokers and Heavy Smokers

**Figure 1:**
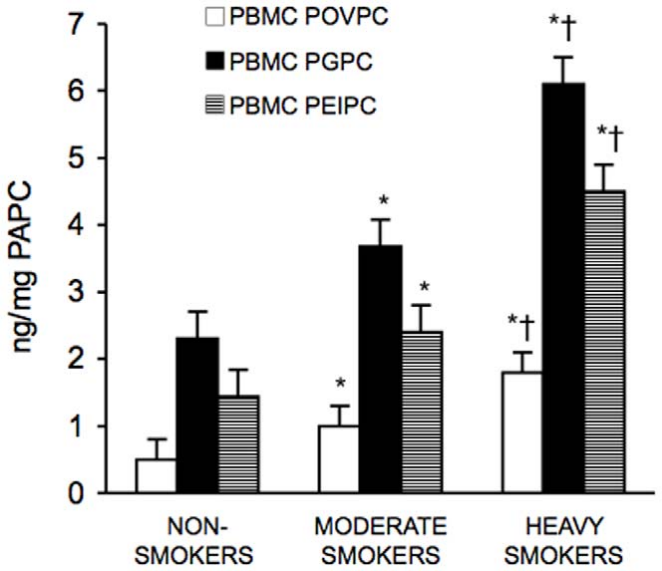
Concentrations of oxidized phospholipids in peripheral blood mononuclear cells (PBMC). Results are the means±SD of measurements performed in triplicate and are expressed in ng/mg PAPC. *p<0.01 versus non-smokers; †p<0.01 versus moderate smokers. PAPC = 1-palmitoyl-2-arachidonyl-sn-glycero-3-phosphorylcholine; PEIPC = 1-palmitoyl-2-(5,6-epoxyisoprostane E2)-sn-glycero-3-phosphocholine; POVPC = 1-palmitoyl-2-(5-oxovaleroyl)-sn-glycero-3-phosphorylcholine; PGPC = 1-palmitoyl-2-glutaroyl-sn-glycero-3-phosphorylcholine.

Figure 1 shows the mean concentrations of PEIPC, POVPC and PGPC in PBMC derived from non-smokers, moderate smokers and heavy smokers. PEIPC, POVPC and PGPC resulted significantly higher in PBMC of moderate smokers and heavy smokers than in non-smokers (p<0.01). PEIPC, POVPC and PGPC were higher in heavy smokers than in moderate smokers (p<0.01).

### Expression of IkB-Alpha, p47phox, p65, Nrf2, HO-1, gGCS, Activation of NF-kB and Expression of IL-6 in PBMC of Non-Smokers, Moderate Smokers and Heavy Smokers

**Figure 2:**
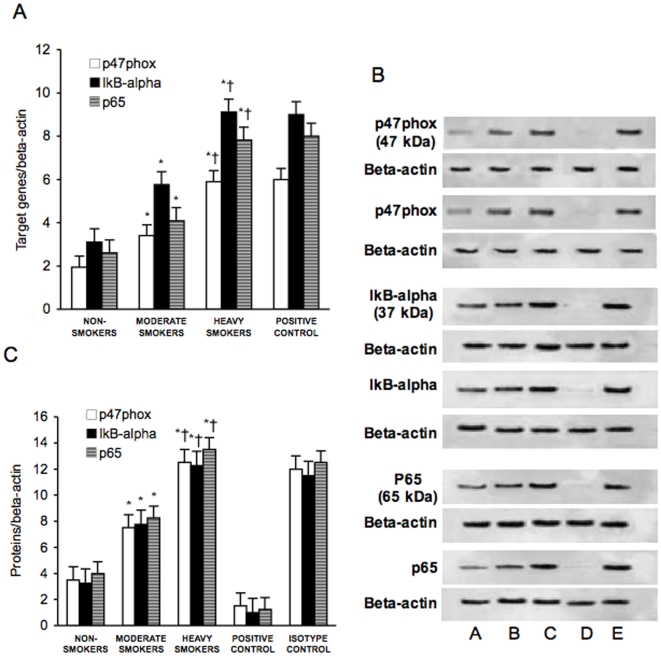
p47phox, IkB-alpha and p65 mRNA and protein expression in peripheral blood mononuclear cells. mRNA (A) was analyzed by quantitative Real-Time PCR. Normalized gene expression levels were given as the ratio between the mean value for the target gene and that for the beta-actin in each sample. Figure shows two representative western blot analysis for p47phox, IkB-alpha and p65 (B) and the average quantification obtained by densitometric analysis of all the samples (C). Data on Western blot analysis are expressed as the density ratio of target to control (Beta-actin) in arbitrary units ×10. Results are the means±SD of measurements performed in triplicate. *p<0.01 versus non-smokers; †p<0.01 versus moderate smokers. A = Non-smokers; B = Moderate smokers; C = Heavy smokers; D = Isotype control; E = Positive control.

**Figure 3:**
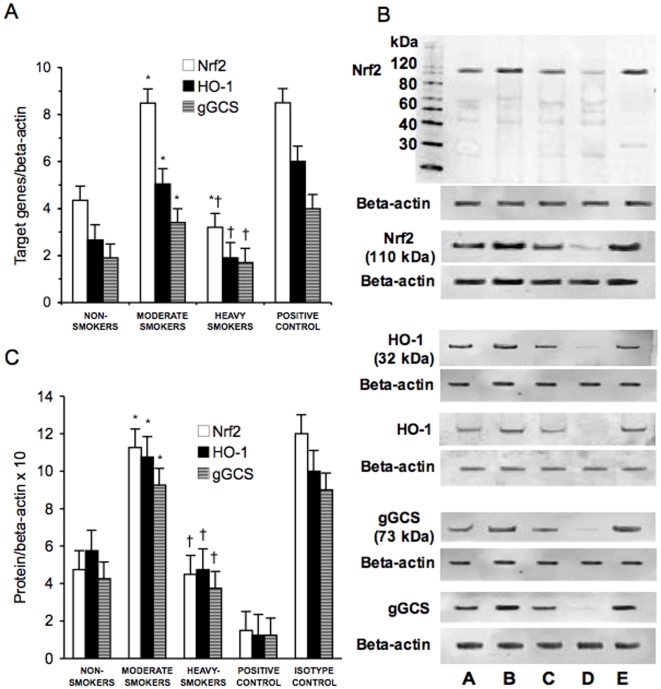
Nrf2, HO-1 and gGCS mRNA and protein expression in peripheral blood mononuclear cells. mRNA (A) was analyzed by quantitative Real-Time PCR. Normalized gene expression levels were given as the ratio between the mean value for the target gene and that for the beta-actin in each sample. Figure shows two representative western blots analysis for Nrf2, HO-1 and gGCS (B) and the average quantification obtained by densitometric analysis of all the ssmples (C). Data on Western blot analysis are expressed as the density ratio of target to control (Beta-actin) in arbitrary units ×10. Results are the means±SD of measurements performed in triplicate. *p<0.01 versus non-smokers; †p<0.01 versus moderate smokers. Nrf2 = NF-E2-related factor 2; HO-1 = heme oxygenase; gGCS = gamma-glutamylcysteine synthetase; A = Non-smokers; B = Moderate smokers; C = Heavy smokers; D = Isotype control; E = Positive control.

Figure 2 and 3 show the mRNA and protein expression of IkB-alpha, p47phox, p65, Nrf2, HO-1 and gGCS in PBMC derived from non-smokers, moderate smokers and heavy smokers. As previously demonstrated, figure 3 B also shows that Nrf2 migrated at 110 kDa in spite of its lower molecular weight [26]. PBMC expression of IkB-alpha, p47phox, p65 and of Nrf2, HO-1 and gGCS was significantly higher in moderate smokers than in non-smokers (p<0.01). In heavy smokers only IkB-alpha, p47phox and p65 expression was higher than in moderate smokers (p<0.01), while Nrf2, HO-1 and gGCS expression was similar to non-smokers (p<0.01).

**Figure 4:**
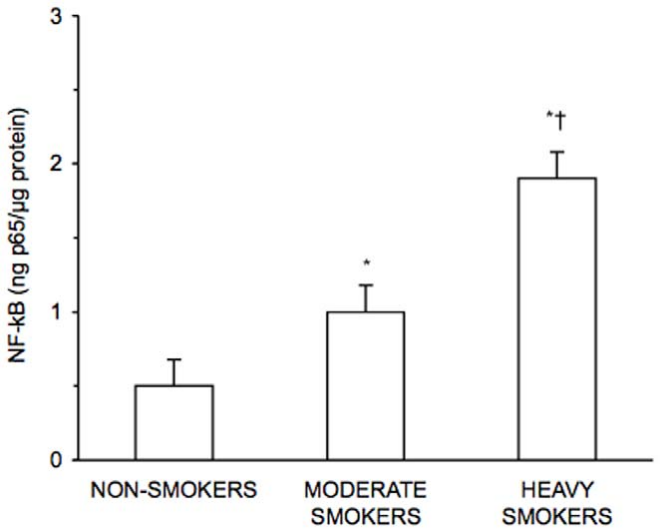
Concentrations of nuclear NF-kB in peripheral blood mononuclear cells. NF-kB was measured in nuclear extracts of PBMC utilizing recombinant p65 as a reference. Results are the means±SD of measurements performed in triplicate. *p<0.01 versus non-smokers; †p<0.01 versus moderate smokers. NF-kB = nuclear factor-kB.

**Figure 5:**
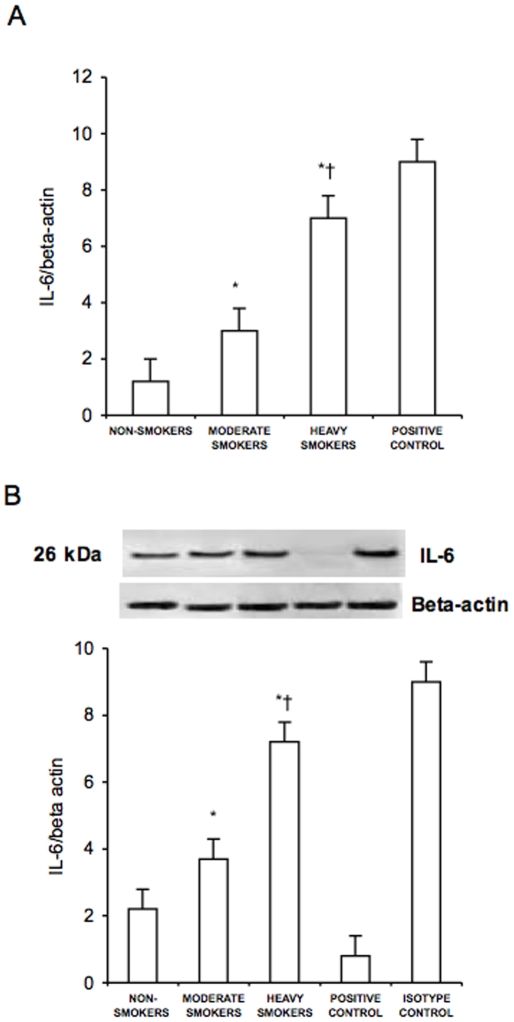
Interleukin-6 (IL-6) mRNA and protein expression in peripheral blood mononuclear cells. mRNA (A) was analyzed by quantitative Real-Time PCR. Normalized gene expression levels were given as the ratio between the mean value for the target gene and that for the beta-actin in each sample. Figure shows a representative western blot analysis for IL-6 and the average quantification obtained by densitometric analysis of all the samples (B). Data on Western blot analysis are expressed as the density ratio of target to control (Beta-actin) in arbitrary units ×10. Results are the means±SD of measurements performed in triplicate. *p<0.01 versus non-smokers; †p<0.01 versus moderate smokers.

**Figure 6:**
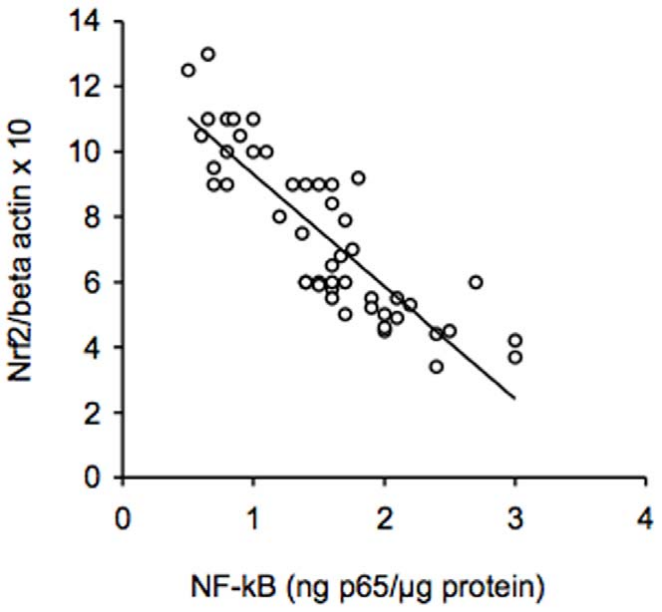
Correlation between the concentration of nuclear NF-kB and protein expression of Nrf2 in peripheral blood mononuclear cells of moderate and heavy smokers. NF-kB was measured in nuclear extracts of PBMC utilizing recombinant p65 as a reference. Data on protein expression of Nrf2 derived from Western blot analysis and are expressed as the density ratio of target to control (Beta-actin) in arbitrary units ×10. NF-kB = nuclear factor-kB; Nrf2 = NF-E2-related factor 2.

Figures 4 and 5 show the concentrations of nuclear NF-kB and the expression of IL-6 in PBMC of non-smokers, moderate smokers and heavy smokers. Nuclear NF-kB concentrations and IL-6 expression were higher in moderate smokers and heavy smokers than in non-smokers (p<0.01). NF-kB values and IL-6 expression resulted significantly higher in heavy smokers than in moderate smokers (p<0.01). In moderate and heavy smokers a negative correlation was found between Nrf2 protein expression and the concentration of nuclear NF-kB using recombinant p65 as a reference (r = −0.71, p<0.001) (Figure 6).

### NADPH-Oxidase Activation and Generation of ROS Are Required for oxPAPC-Dependent Expression of Nrf2

**Figure 7:**
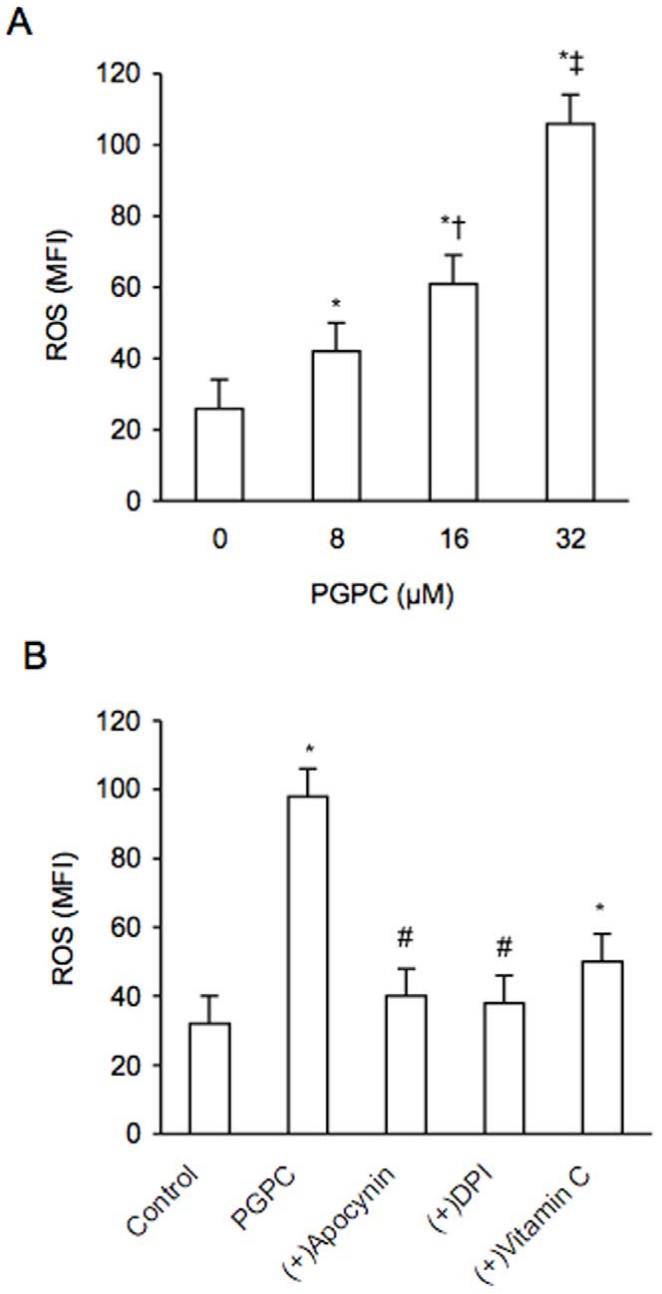
Effect of NADPH-oxidase inhibitors and vitamin C on reactive oxygen species (ROS) induced by PGPC in peripheral blood mononuclear cells. The concentration of PGPC ranged from 0 to 32 µM (A). The inhibitors of NADPH-oxidase apocynin and diphenyliodonium (DPI) were used at a concentration of 50 µM; vitamin C at a concentration of 400 µM (B). Data are expressed as mean fluorescence intensity (MFI) and represent the means±SD of measurements performed in triplicate in six different occasions. *p<0.01 versus 0 µM and control; †p<0.01 versus 8 µM; ‡<0.01 versus 16 µM; #<001 versus PGPC. PGPC = 1-palmitoyl-2-glutaroyl-sn-glycero-3-phosphorylcholine.

**Figure 8:**
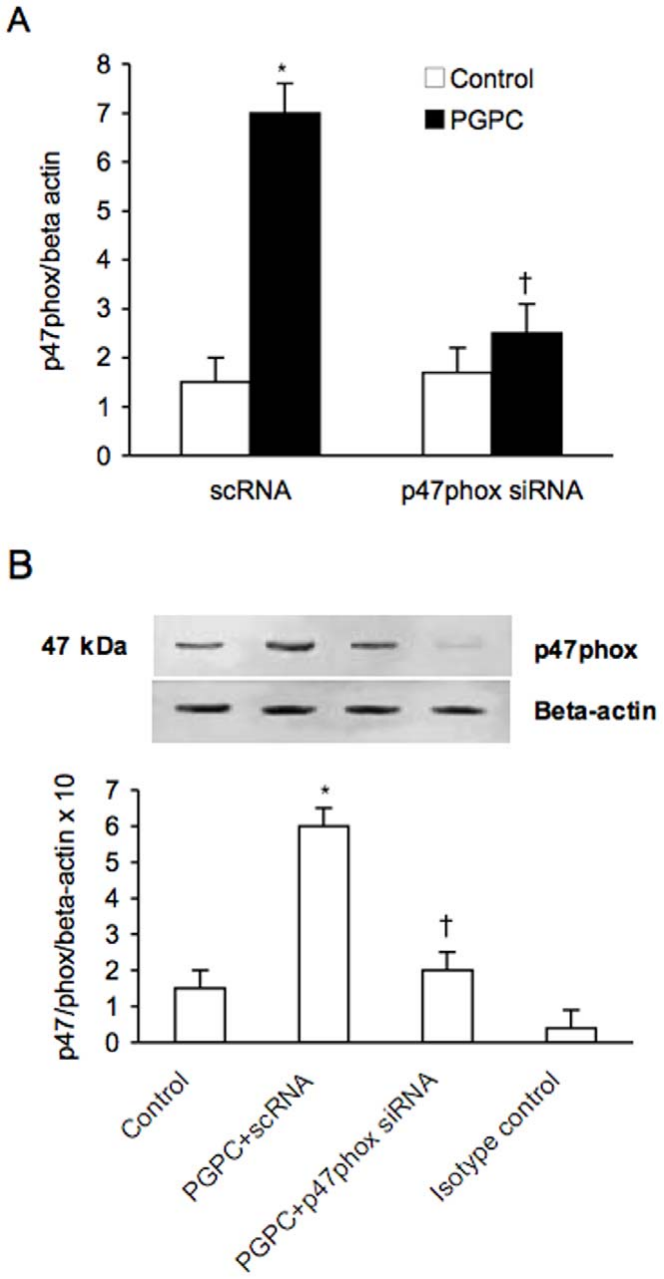
Effect of small interfering (si) and scrambler (sc) RNA against p47phox on PGPC-dependent expression of p47phox in peripheral blood mononuclear cells. mRNA (A) was analyzed by quantitative Real-Time PCR. Normalized gene expression levels were given as the ratio between the mean value for the target gene and that for the beta-actin in each sample.. Figure shows a representative western blot analysis for p47phox and the average quantification obtained by densitometric analysis of all the samples (B). Data on Western blot analysis are expressed as the density ratio of target to control (Beta-actin) in arbitrary units ×10. Results are the means±SD of measurements performed in triplicate in six different occasions. *p<0.01 versus control; †p<0.01 versus PGPC; ‡<0.01 versus scRNA. PGPC = 1-palmitoyl-2-glutaroyl-sn-glycero-3-phosphorylcholine.

**Figure 9:**
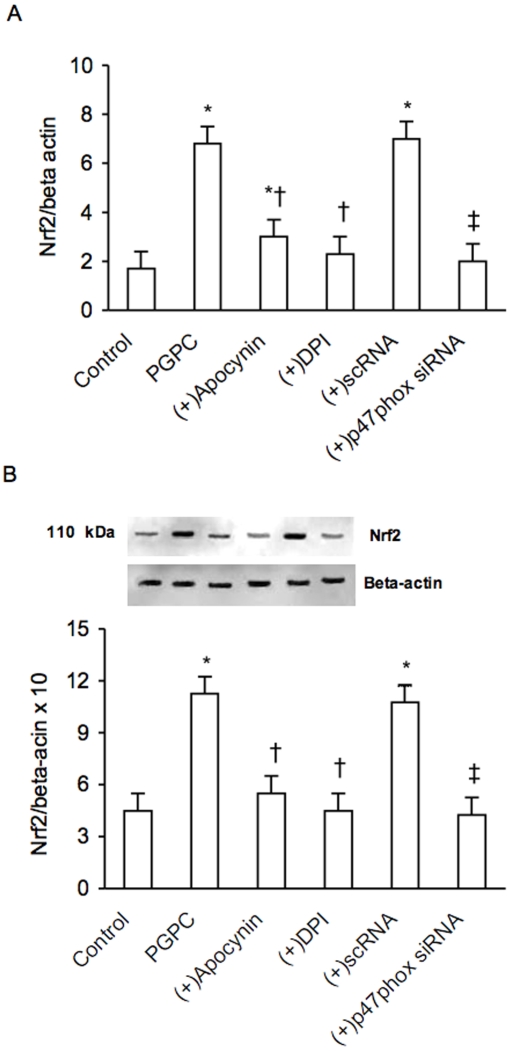
Effect of small interfering (si) and scrambler (sc) RNA against Nrf2, apocynin and diphenyliodonium (DPI) on PGPC-dependent expression of Nrf2 in peripheral blood mononuclear cells. PGPC, apocynin and DPI were used at a concentration of 16 and 50 µM respectively. mRNA (A) was analyzed by quantitative Real-Time. Normalized gene expression levels were given as the ratio between the mean value for the target gene and that for the beta-actin in each sample. Figure shows a representative western blot analysis for Nrf2 and the average quantification obtained by densitometric analysis of all the samples (B). Data on Western blot analysis are expressed as the density ratio of target to control (Beta-actin) in arbitrary units ×10. Results are the means±SD of measurements performed in triplicate in six different occasions. *p<0.01 versus control; †p<0.01 versus PGPC; ‡<0.01 versus scRNA. Nrf2 = NF-E2-related factor 2; PGPC = 1-palmitoyl-2-glutaroyl-sn-glycero-3-phosphorylcholine.

Increasing amounts of POVPC, PGPC and PEIPC determined a dose-dependent, significant rise of ROS in PBMC derived from healthy volunteers as evaluated by flow cytometry (data not shown). Figure 7 shows the dose-dependent effect of PGPC (in a range from 0 to 32 µM) on ROS generation. The PGPC-dependent generation of ROS was inhibited by the NADPH inhibitors apocynin and DPI (p<0.01) and by vitamin C (Figure 7). Furthermore siRNA-mediated knockdown of p47phox determined a significant reduction of PGPC-dependent expression of p47pho***x*** (Figure 8). The reduction of ROS induced by the inhibitors of NADPH oxidase and by p47phox siRNA significantly reduced the expression of Nrf2 (p<0.01) (Figure 9), meaning that generation of ROS are required for oxPAPC-dependent expression of Nrf2.

### Nrf2 Is Required for ox-PAPC-Stimulated HO-1 and gGCS Expression

**Figure 10:**
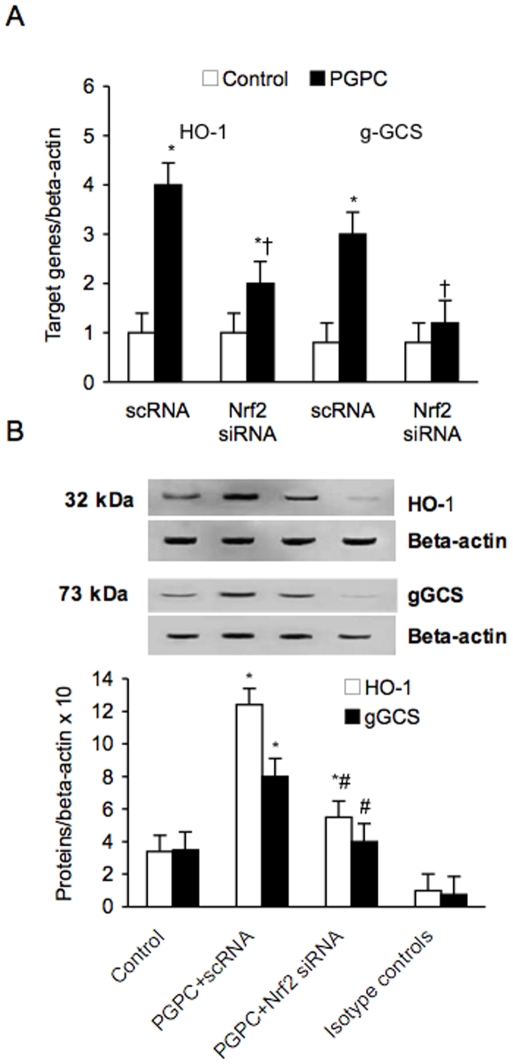
Effect of small interfering (si) and scrambler (sc) RNA against Nrf2 on PGPC-dependent expression of HO-1 and gGCS in peripheral blood mononuclear cells. mRNA (A) was analyzed by quantitative Real-Time PCR. Normalized gene expression levels were given as the ratio between the mean value for the target gene and that for the beta-actin in each sample. Figure shows a representative western blot analysis for HO-1 and gGCS and the average quantification obtained by densitometric analysis of all the samples (B). Data on Western blot analysis are expressed as the density ratio of target to control (Beta-actin) in arbitrary units ×10. Results are the means±SD of measurements performed in triplicate in six different occasions. *p<0.01 versus control; †p<0.01 versus PGPC; #<0.01 versus PGPC+scRNA. Nrf2 = NF-E2-related factor 2; PGPC = 1-palmitoyl-2-glutaroyl-sn-glycero-3-phosphorylcholine; HO-1 = heme oxygenase; gGCS = gamma-glutamylcysteine synthetase.

To determine whether Nrf2 is required for PGPC-stimulated HO-1 and gGCS expression, we used siRNA to knockdown the expression of Nrf2. Nrf2 siRNA reduced significantly Nrf2 mRNA expression (data not shown). Figure 10 shows that the knockdown of Nrf2 was associated to a significant reduction of HO-1 and gGCS expression induced by PGPC (p<0.01).

### NF-kB Activation Inhibits oxPAPC-Dependent Expression of Nrf2 in PBMC Derived from Healthy Donors

**Figure 11:**
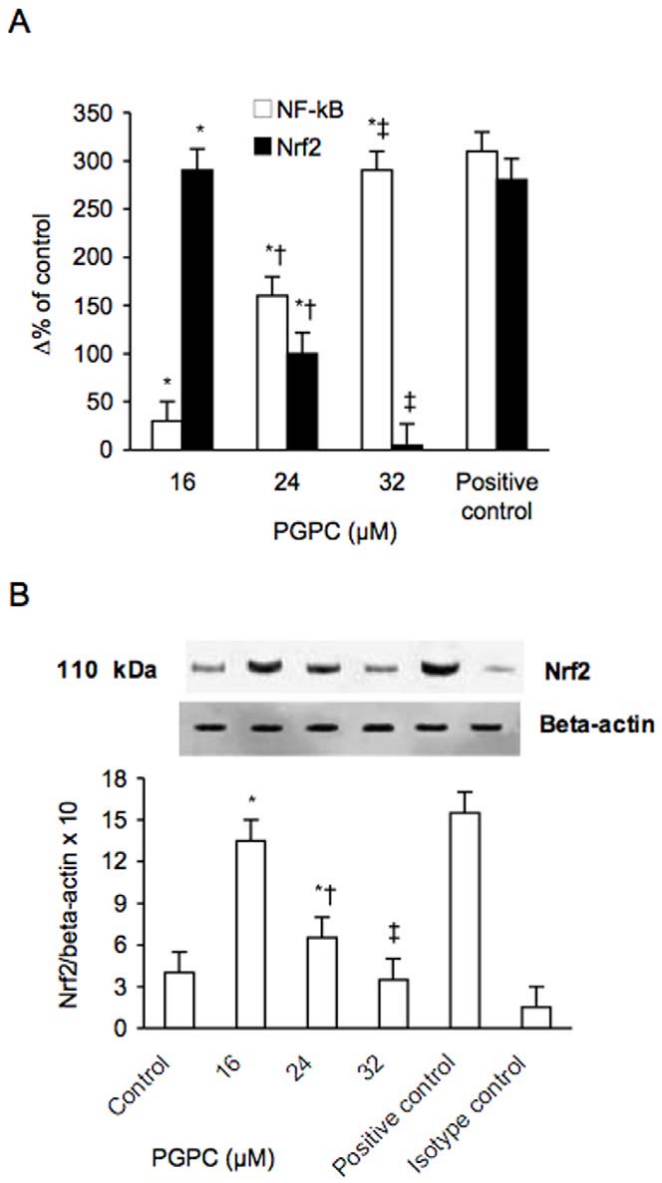
Effect of PGPC on nuclear NF-kB activation and Nrf2 expression in peripheral blood mononuclear cells. NF-kB (A) was analyzed as described in materials and methods; Nrf2 mRNA (A) was analyzed by quantitative Real-Time PCR. Normalized gene expression levels were given as the ratio between the mean value for the target gene and that for the beta-actin in each sample. Figure shows a representative western blot analysis for Nrf2 and the average quantification obtained by densitometric analysis of all the samples (B). Data on Western blot analysis are expressed as the density ratio of target to control (Beta-actin) in arbitrary units ×10. Results are the means±SD of measurements performed in triplicate in six different occasions. *p<0.01 versus control; †p<0.01 versus 16 µM PGPC; ‡ p<0.01versus 24 µM PGPC. NF-kB = nuclear factor-kB; Nrf2 = NF-E2-related factor 2.

**Figure 12:**
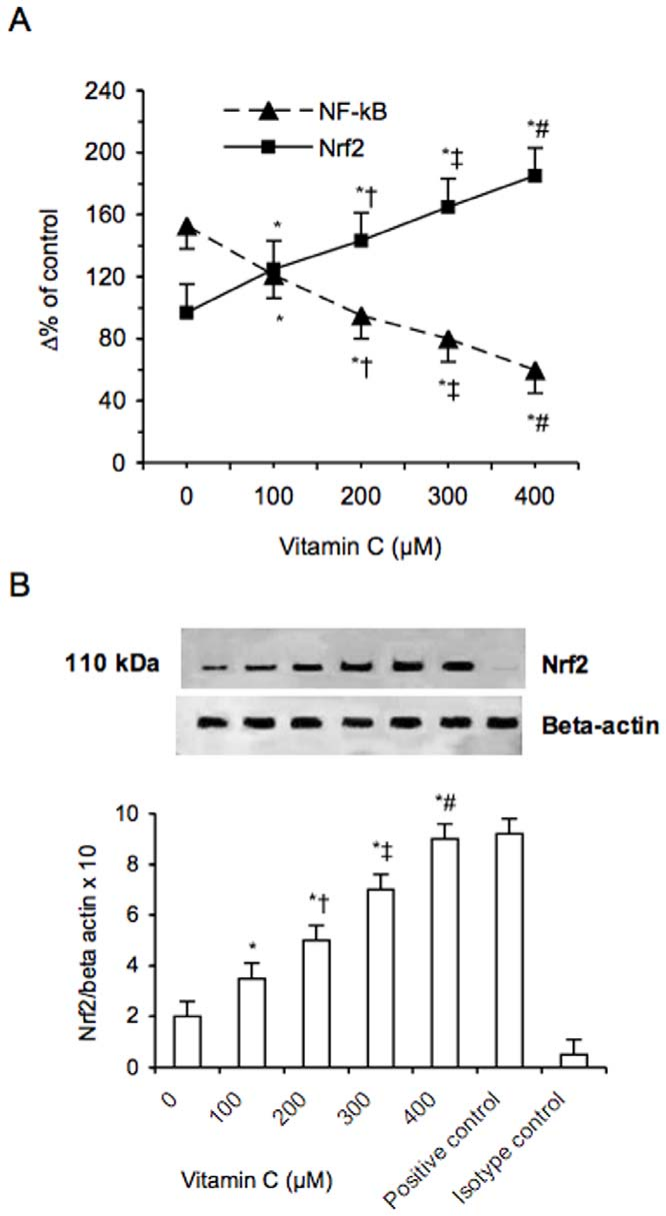
Effect of increasing amounts of vitamin C on PGPC-dependent activation of nuclear NF-kB and Nrf2 expression in peripheral blood mononuclear cells. PGPC was used at a concentration of 24 µM. NF-kB (A) was analyzed as described in materials and methods; Nrf2 mRNA (A) was analyzed by quantitative Real-Time PCR. Normalized gene expression levels were given as the ratio between the mean value for the target gene and that for the beta-actin in each sample. Figure shows a representative western blot analysis for Nrf2 and the average quantification obtained by densitometric analysis of all the samples (B). Data on Western blot analysis are expressed as the density ratio of target to control (Beta-actin) in arbitrary units ×10. Results are the means±SD of measurements performed in triplicate in six different occasions. *p<0.01 versus control; †p<0.01 versus 100 µM vitamin C; ‡p<0.01 versus 200 µM vitaminC; #p<0.01 versus 300 µM vitamin C. PGPC = 1-palmitoyl-2-glutaroyl-sn-glycero-3-phosphorylcholine; NF-kB = nuclear factor-kB, Nrf2 = Nrf2 = NF-E2-related factor 2.

Increasing amounts of PGPC (in a range from 0 to 32 µM) dose-dependently increased NF-kB activation in PBMC derived from healthy donors (p<0.01) (Figure 11). At these concentrations the increase of NF-kB activation was associated to a progressive decrease of Nrf2 expression. Vitamin C dose-dependently attenuated this phenomenon (Figure 12).

**Figure 13:**
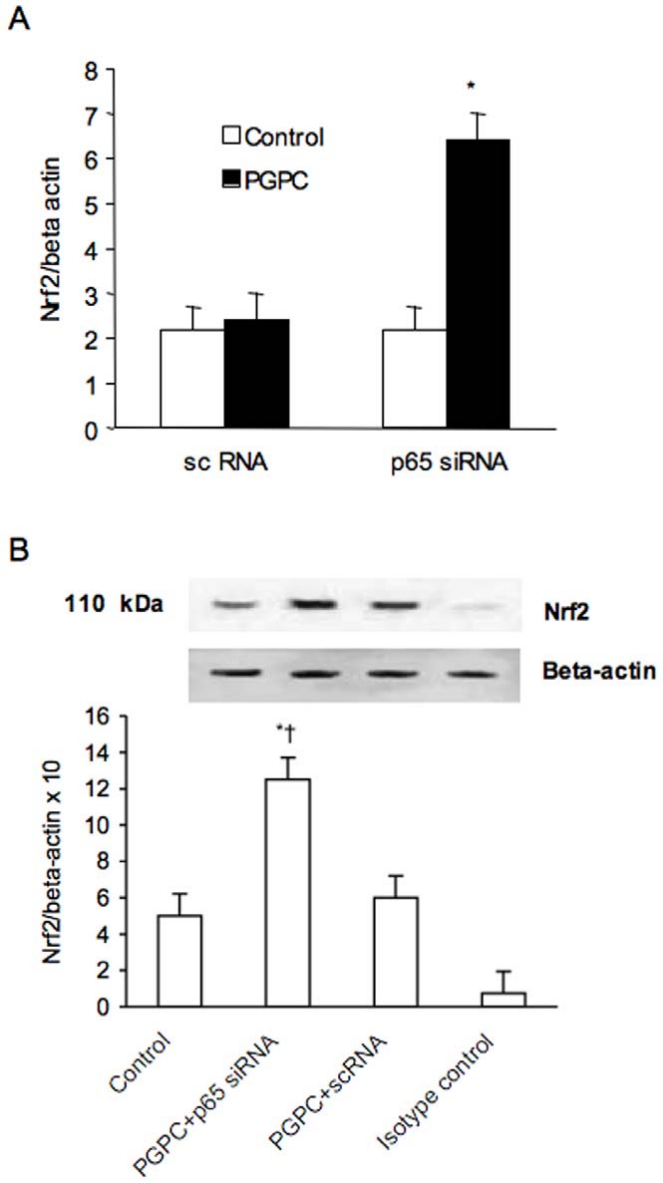
Effect of small interfering (si) and scrambler (sc) RNA against p65 on PGPC-dependent expression of Nrf2 in peripheral blood mononuclear cells. PGPC was used at a concentration of 32 µM. mRNA (A) was analyzed by quantitative Real-Time PCR. Normalized gene expression levels were given as the ratio between the mean value for the target gene and that for the beta-actin in each sample. Figure shows a representative western blot for Nrf2 and the average quantification obtained by densitometric analysis of all the samples (B). Data on Western blot analysis are expressed as the density ratio of target to control (Beta-actin) in arbitrary units ×10. Results are the means±SD of measurements performed in triplicate in six different occasions. *p<0.01 versus control; †p<0.01 versus scRNA. PGPC = 1-palmitoyl-2-glutaroyl-sn-glycero-3-phosphorylcholine; Nrf2 = NF-E2-related factor 2.

To determine why nuclear NF-κB increase caused a fall in Nrf2 expression we used siRNA to knockdown the expression of NF-κB/p65. With the highest concentration of PGPC added (32 µM), p65 siRNA increased about three times the expression of Nrf2 (Figure 13).

## Discussion

Overwhelming evidence shows that cigarette smoking is an important risk factor for atherosclerosis [9]. However the underlying factors of this deleterious effect are not fully understood. In this study we showed for the first time that in healthy young smokers, cigarette smoking is associated with increased PGPC, POVPC and PEIPC levels in PBMC. Our results are in line with a series of other studies demonstrating that cigarette smoking induces systemic oxidative stress (reviewed in 27). The increase of these oxPAPC in PBMC was associated with the activation of the Nrf2/ARE pathway, that is involved in the induction of genes coding for a number of antioxidants [15]. The activation of this pathway, however, was evident only in moderate smokers, i.e. in subjects smoking no more than 10 cigarettes per day, while in heavy smokers, i.e. in subjects smoking more than 25 cigarettes per day, the Nrf2/ARE pathway was not activated or even repressed. The reason for this discrepancy is unclear, but these results are supported by our *in vitro* findings showing that at the highest concentrations of PGPC, the Nrf2/ARE pathway was no longer stimulated or even reduced. OxPAPC were previously found to increase ROS production via NADPH-oxidase activation when added to cultured cells [14]. In *in vitro* studies we too showed that PGPC, POVPC and PEIPC singly and dose-dependently rose the generation of ROS in PBMC derived from healthy donors and that this increase was related to the activation of NADPH-oxidase. The oxPAPC-induced increase of ROS was associated to an induction of Nrf2 expression that was inhibited when the cells were incubated with NADPH-oxidase inhibitors and with siRNA-mediated knockdown of p47phox. It is likely that this mechanism is operating also *in vivo* since the expression of p47phox, that reasonably reflects the activity of NADPH-oxidase, was much higher in the PBMC of heavy smokers who had the highest concentrations of cellular PGPC, POVPC and PEIPC, than in moderate smokers or non-smokers. Furthermore the fact that the concentration of GSH in PBMC was much lower in heavy smokers than in moderate smokers indicates that oxidative stress was at the most in the former group. Since it has been suggested that the Nrf2/ARE pathway is crucial for the regulation of intracellular redox state [16] and oxidative stress is one of the most potent stimuli for Nrf2/ARE pathway activation [28], a first conclusion of this study is that heavy smokers, contrary to moderate smokers, do not appropriately react in terms of Nrf2/ARE activation to the intracellular oxidative stress. This inadequacy of response was also seen in *in vitro* studies where the highest degree of oxidative stress did not cause the activation of Nrf2/ARE pathway. Under different experimental conditions our results agree with a series of recent studies showing a decline of Nrf2 expression in whole lung tissue and alveolar macrophages of patients with pulmonary emphysema [29] and in pulmonary macrophages of current smokers and patients with chronic obstructive pulmonary disease [30], [31] a pathological situation in which oxidant-antioxidant imbalance is strongly implicated [32], [33].

The results of this study also demonstrate that contrary to the Nrf2/ARE pathway, there was a progressive activation of NF-kB in the PBMC of moderate smokers and heavy smokers compared to non-smokers and that this activation was associated to a functional consequence, i.e. the increase of circulating and PBMC IL-6. A huge amount of experimental data supports the activation of the transcription factor NF-kB as a key redox-sensitive event associated with vascular dysfunction (reviewed in 7). So the progressive increase of NF-kB from non-smokers to heavy smokers may be related to the oxPAPC-induced generation of ROS. Since NF-kB intervenes in the transcription of a large number of inflammatory genes and in particular of IL-6 [8], that induces CRP in the liver [34], the increase in CRP of smokers may be related to NF-kB activation. The levels of CRP, even though in the normal range, were much higher (about three times) in heavy smokers than in moderate smokers. Of course this may derive from the higher number of cigarettes the group of heavy smokers smoked per day and therefore from the greater oxidative stress. In moderate smokers, but not in heavy smokers however, there may be the counteracting effect of the Nrf2/ARE apparatus. Nrf2's regulation of cellular GSH and other antioxidants has been shown to be critical for a correct modulation of NF-kB activation [35]. Moreover it has recently been shown that Nrf2 is a critical regulator of NF-kB activation both by modulating Ik-B degradation and MyD88-dependent and -independent signalling [35].

The *in vitro* results on NF-kB are in line with the data we obtained *in vivo*. Increasing amounts of PGPC, in fact, dose-dependently increased the activation of NF-kB in PBMC derived from healthy donors, while at the highest concentrations Nrf2 was no longer stimulated. Vitamin C was found to attenuate this phenomenon suggesting that oxidative stress plays a major role in NF-kB activation and in inappropriate Nrf2 expression. Even though these results have to be confirmed *in vivo* and therefore do not allow any conclusion, they are in line with the findings of Waltenberger et al [36] who found that vitamin C reversed smoking-induced monocyte dysfunction *in vivo*.

The reasons for this unexpected result on Nrf2 are unclear. Recent data have shown that NF-kB p65 subunit repressed the Nrf2/ARE pathway at transcriptional level [17]. In particular, in the cells where NF-kB and Nrf2 were simultaneously activated, p65 unidirectionally antagonized the transcriptional activity of Nrf2. In our study in order to determine why NF-kB activation caused a fall in Nrf2 expression, we used siRNA to knockdown the expression of NF-kB/p65. With the highest concentration of PGPC added (32 µM), p65 siRNA increased about three times the expression of Nrf2 suggesting that the Nrf2 repression may be related to the p65 antagonism. Of course on the basis of these results we cannot exclude that Nrf2 decline in heavy smokers may also be related to other mechanisms and in particular to decreased Nrf2 protein stability as recently demonstrated in patients with COPD [31].

In conclusion the results of this study show that cigarette smoking is associated with increased oxPAPC in PBMC of young healthy smokers with no additional risk factors for atherosclerosis. This means that water-soluble components of cigarette smoke reach the systemic circulation and can promote oxidative stress not only of blood cells but probably also of vasculature cells. Increased oxPAPC and in particular PGPC, POVPC and PEIPC were associated with the activation of the Nrf2/ARE pathway only in moderate smokers and not in heavy smokers, while NF-kB was progressively more activated from non-smokers to heavy smokers. This investigation revealed that NF-kB p65 may participate in the negative regulation of Nrf2/ARE signalling, and might provide a new insight into a possible role of NF-kB in suppressing the expression of antioxidant genes, favouring inflammation.
